# Genome Sequences of Three SARS-CoV-2 ORF7a Deletion Variants Obtained from Patients in Hong Kong

**DOI:** 10.1128/MRA.00251-21

**Published:** 2021-04-15

**Authors:** Herman Tse, Sally Cheuk-Ying Wong, Ka-Fai Ip, Vincent Chi-Chung Cheng, Kelvin Kai-Wang To, David Christopher Lung, Garnet Kwan-Yue Choi

**Affiliations:** a Department of Pathology, Hong Kong Children’s Hospital, Hong Kong Special Administrative Region, China; b Department of Microbiology, Queen Mary Hospital, Hong Kong Special Administrative Region, China; c State Key Laboratory of Emerging Infectious Diseases, Carol Yu Centre for Infection, Department of Microbiology, Li Ka Shing Faculty of Medicine, The University of Hong Kong, Hong Kong Special Administrative Region, China; d Department of Microbiology, The University of Hong Kong, Hong Kong Special Administrative Region, China; e Department of Pathology, Queen Elizabeth Hospital, Hong Kong Special Administrative Region, China; Queens College CUNY

## Abstract

We report the genome sequences of severe acute respiratory syndrome coronavirus 2 (SARS-CoV-2) strains from the clinical samples of three coronavirus disease 2019 (COVID-19) patients in Hong Kong. All the genome sequences showed a 370-nucleotide deletion resulting in the complete loss of ORF7a.

## ANNOUNCEMENT

Severe acute respiratory syndrome coronavirus 2 (SARS-CoV-2) is a novel member of the genus *Betacoronavirus* in the family *Coronaviridae* and the causative agent of coronavirus disease 2019 (COVID-19). More than 100 million cases of infection had been reported globally by the end of January 2021 (1). In this report, we present the genome sequences of SARS-CoV-2 strains from 3 COVID-19 patients in Hong Kong obtained during investigation of a local cluster in mid-January 2021. The study protocol was approved by the institutional review board of The University of Hong Kong/Hospital Authority Hong Kong West Cluster (UW 13-372).

Saliva specimens were collected by patients as described previously (2). Total nucleic acid extraction was performed using the MagNA Pure 96 DNA and viral NA small-volume kit (Roche, Switzerland). Tiling PCR for specific preamplification of the SARS-CoV-2 genome was performed using the ARTIC v3 primer set (https://github.com/artic-network/artic-ncov2019/tree/master/primer_schemes/nCoV-2019/V3) according to the specified protocol (https://www.protocols.io/view/ncov-2019-sequencing-protocol-v3-locost-bh42j8ye). Library preparation for multiplexed sequencing, with up to 32 samples per run, was performed using IDT for Illumina DNA/RNA unique dual (UD) indexes and the Illumina DNA prep kit (California, USA). Sequencing of the pooled library was performed on an iSeq 100 sequencer (Illumina) to obtain paired-end 150-bp reads.

Fastp version 0.21.0 (3) was used for quality control and preprocessing of demultiplexed read data. Subsequent processing was performed according to the Utah Department of Health ARTIC/Illumina Bioinformatic Workflow (https://github.com/CDCgov/SARS-CoV-2_Sequencing/tree/master/protocols/BFX-UT_ARTIC_Illumina), except for the use of bwa-mem2 instead of bwa for mapping reads. Briefly, reads were mapped to a reference SARS-CoV-2 genome sequence (GenBank accession number MN908947.3) with bwa-mem2 version 2.0 (4), and primer trimming was performed using iVar version 1.3 (5). Variant calling and consensus sequence generation were performed using SAMtools version 1.10 (6). The genome assembly was visualized and examined using Tablet version 1.20.12.24 (7).

The sequencing statistics and genomic features of the genome assemblies are summarized in Table 1. All 3 strains belonged to the PANGO lineage B.1.36, as classified by Pangolin version 2.3.0 (lineages version 2021-02-21) (https://github.com/cov-lineages/pangolin). In comparison, most local cases in Hong Kong since September 2020 have been caused by viruses from lineage B.1.36.27 (https://cov-lineages.org/lineages/lineage_B.1.36.27.html). Notably, the present sequences contained a 370-nucleotide deletion spanning positions 27387 to 27756 of the reference SARS-CoV-2 genome. This major deletion was confirmed independently by long-range PCR and Sanger sequencing (with primers 5′-AACTCGTAATCGGAGCTGTGA-3′ and 5′-TGTCATTCTCCTAAGAAGCTATTAAA-3′). As a result, the entire ORF7a and the original stop codon for ORF6 were lost. The latter resulted in an extension of the Orf6 protein by 2 amino acids. Furthermore, ORF7b was potentially extended in the 5′ direction by 27 nucleotides due to the occurrence of an in-frame start codon.

**TABLE 1:** Sequencing statistics and features of the SARS-CoV-2 genome assemblies^*a*^

Strain	Sequence length (nucleotides)	G+C content (%)	Mean read depth^*b*^ (×)	Genomic coverage^*b*^ (%)	PANGO lineage	Specific nucleotide (and amino acid) substitution(s)^*b*^^,^^*c*^	Other genomic feature^*b*^
HKCH_932	29,428	38.0	1,107.4	98.8	B.1.36	C11563T, C17590T	370-nucleotide deletion from reference genome position 27387 to 27756
HKCH_933	29,428	38.0	1,046.6	98.8	B.1.36	370-nucleotide deletion from reference genome position 27387 to 27756
HKCH_934	29,428	38.0	1,176.5	98.8	B.1.36	C11563T	370-nucleotide deletion from reference genome position 27387 to 27756

a Nucleotide (and amino acid) substitutions common to all three strains (mapped against the genome sequence of reference SARS-CoV-2 strain Wuhan-Hu-1; GenBank accession number MN908947.3) include C241T, C3037T, C4276T, C5339T (nsp3 P874S), C5812T, C6027T (nsp3 P1103L), C10039T, C10456T, C14408T (nsp12 P323L), C18877T, C19572T, C20844T, C22444T, T22882G (spike N440K), A23403G (spike D614G), G23587T (spike Q675H), G25563T (orf3a Q57H), G25785T (orf3a W131C), C26625T, C26735T, G28690T (nucleocapsid L139F), and C28854T (nucleocapsid S194L). The nucleotide substitution is synonymous or in a noncoding region if no accompanying amino acid substitution is shown.

b When mapped against the genome sequence of reference SARS-CoV-2 strain Wuhan-Hu-1 (GenBank accession number MN908947.3).

c The nucleotide substitution is synonymous or in a noncoding region if no accompanying amino acid substitution is shown.

Various major ORF7a deletions, sometimes alongside other major deletions, have been reported previously (8–10). The repeated reemergence of ORF7a deletion variants may suggest that the accessory protein could impose a fitness cost that exceeds the benefits of its canonical function in certain situations.

### Data availability.

These genome sequences were deposited in GenBank under accession numbers MW691225, MW691226, and MW691227. The raw reads were deposited in SRA under accession numbers SRR13775065, SRR13775066, and SRR13775067.
